# Genetic variants of the human host influencing the coronavirus-associated phenotypes (SARS, MERS and COVID-19): rapid systematic review and field synopsis

**DOI:** 10.1186/s40246-020-00280-6

**Published:** 2020-09-11

**Authors:** Emilio Di Maria, Andrea Latini, Paola Borgiani, Giuseppe Novelli

**Affiliations:** 1 Department of Health Sciences, University of Genova, Genova, Italy; 2 Unit of Medical Genetics, Galliera Hospital, Genova, Italy; 3 Department of Biomedicine and Prevention, Genetics Unit, University of Roma “Tor Vergata”, Roma, Italy; 4 IRCCS Neuromed, Pozzilli (IS), Italy; 5 Department of Pharmacology, School of Medicine, University of Nevada, Reno, NV 89557 USA

**Keywords:** COVID-19, Coronavirus, Genomic biomarker, Human host, Genetic susceptibility, Genetic association, Genotype, Polymorphism

## Abstract

The COVID-19 pandemic has strengthened the interest in the biological mechanisms underlying the complex interplay between infectious agents and the human host. The spectrum of phenotypes associated with the SARS-CoV-2 infection, ranging from the absence of symptoms to severe systemic complications, raised the question as to what extent the variable response to coronaviruses (CoVs) is influenced by the variability of the hosts’ genetic background.

To explore the current knowledge about this question, we designed a systematic review encompassing the scientific literature published from Jan. 2003 to June 2020, to include studies on the contemporary outbreaks caused by SARS-CoV-1, MERS-CoV and SARS-CoV-2 (namely SARS, MERS and COVID-19 diseases). Studies were eligible if human genetic variants were tested as predictors of clinical phenotypes.

An ad hoc protocol for the rapid review process was designed according to the PRISMA paradigm and registered at the PROSPERO database (ID: CRD42020180860). The systematic workflow provided 32 articles eligible for data abstraction (28 on SARS, 1 on MERS, 3 on COVID-19) reporting data on 26 discovery cohorts. Most studies considered the definite clinical diagnosis as the primary outcome, variably coupled with other outcomes (severity was the most frequently analysed). Ten studies analysed HLA haplotypes (1 in patients with COVID-19) and did not provide consistent signals of association with disease-associated phenotypes. Out of 22 eligible articles that investigated candidate genes (2 as associated with COVID-19), the top-ranked genes in the number of studies were *ACE2*, *CLEC4M* (L-SIGN), *MBL*, *MxA* (*n* = 3), *ACE*, *CD209*, *FCER2*, *OAS-1*, *TLR4*, *TNF-α* (*n* = 2). Only variants in *MBL* and *MxA* were found as possibly implicated in CoV-associated phenotypes in at least two studies. The number of studies for each predictor was insufficient to conduct meta-analyses.

Studies collecting large cohorts from different ancestries are needed to further elucidate the role of host genetic variants in determining the response to CoVs infection. Rigorous design and robust statistical methods are warranted.

## Introduction

Infectious diseases are known to accompany human evolution through a complex interaction between the host and the infection [1]. Infectious diseases, by definition, are caused by a single infectious agent. However, even prior to the current molecular genetics era, heritability studies provided the first line of evidence that part of inter-individual differences is attributable to the host genetics profile [2, 3].

The global impact of tuberculosis and HIV infection, the interest in the understanding of the genetic background of infectious disease, coupled with the emerging molecular technologies, lead to an increase of investigations on the role of the human host genetics profile [4]. Most candidate-gene studies were concentrating on respiratory infections. However, these studies provided conflicting results [5].

Several human-tropic coronaviruses (CoVs) constantly circulate in the human population and usually cause mild respiratory disease. Two of these RNA viruses—SARS-CoV-1 and MERS-CoV—had been discovered in 2002 and 2012, respectively, as the cause of severe acute respiratory syndromes. The former emerged in Guangdong province, China, and its spread in China is known as the SARS epidemic. The latter caused an epidemic that began in Saudi Arabia and was limited in the Middle East (thus named Middle East Respiratory Syndrome, MERS) [6].

In December 2019, a new infectious respiratory disease emerged in Wuhan, Hubei province, China. The disease, termed coronavirus disease 19 (COVID-19), rapidly spread from China as a global devastating pandemic [7–9]. At the time we were drafting this manuscript, the impact of the outbreak was still growing worldwide. According to the World Health Organisation, confirmed cases had exceeded 14 million and the number of deaths 600,000 (covid19.who.int—last access July 20, 2020).

As the knowledge of the COVID-19 risk factors has progressed, age has become recognised as one of the predominant determinants of severe outcomes [10, 11], in association with cardiovascular disease and metabolic disorders as major comorbidity factors [12].

The latest findings of seroprevalence in COVID-19 were allowed to estimate a higher prevalence of infection than previously inferred [13]. This is in line with the hypothesis that the high rate of severe clinical manifestations experienced in the initial spread was attributable to vulnerable individuals. The extent to which the susceptibility to the severe clinical course is due to comorbidity or is determined by constitutional factors needs to be elucidated.

Soon after the SARS outbreak in 2003, researchers tried to respond to the question as to whether germline genetic variants influence the occurrence of the clinical syndrome caused by CoVs as well as its variable outcome. At present, this question has begun a priority for human genomics.

The first extensive systematic review addressing host genetic factors implicated in common respiratory tract infectious diseases is dated after the SARS and MERS outbreaks. Pooled analysis of respiratory infections revealed a significant association with the rs2070874 of the *IL4* gene and additional genetic risk factors for tuberculosis. The study implemented a formal assessment of the risk of bias and concluded that almost 95% of eligible studies were affected by a strong risk of bias or confounding [14].

Immune response is the primary arm which should be explored in infectious diseases. Seminal studies on human Mendelian disorders causing deficiencies of the immune response to viruses (reviewed in Casanova JL et al. 2020 [15]) provided the background to investigate the influence of germline genetic variations in other, much more common, sporadic infectious diseases. According to the current paradigm for the complex disorder, the model to explain the genetic susceptibility to infection is not Mendelian—but may be either monogenic or polygenic—and postulates that any single genetic biomarker may be associated with a risk with low to very low effect.

CoVs bind their cellular receptors using the homotrimeric spike glycoprotein (S1 subunit and S2 subunit in each spike monomer) on the envelope. Such binding triggers a cascade of events leading to the fusion between the cell and viral membranes for cell entry. X-ray crystallography modelling elucidated the structure of the SARS-CoV-2 receptor-binding motif which binds the human Angiotensin 1 Converting Enzyme 2 (ACE2), demonstrating that the binding mode of the SARS-CoV-2 is nearly identical to that observed in previously determined CoV-receptor complex structure [16].

Since it has been demonstrated that the SARS-CoV-2 spike protein interacts with human ACE2 [16, 17], a large number of studies focused on the *ACE2* gene to investigate the hypothesis that variability in ACE2 structure and expression is related to different susceptibility to COVID-19 (recently reviewed by Devaux CA et al. [18]). The variability of *ACE2*, as well as of *TMPRSS2* which promotes SARS-CoV-2 cellular entry, was already explored comparing public data from the population of different ancestries, providing the rationale for investigations on patients [19–21].

COVID-19 is a new disease and the current pandemic is incommensurable with other CoV-related clinical conditions. Remarkably, the SARS-CoV-2 infection occurs on a global scale with no influence from acquired immunity, either from vaccines or previous exposures. In turn, this tragic experience is an unprecedented opportunity to explore the genetic determinants of an emerging infection. Therefore, the human genetics community should endorse an extraordinary scientific effort to comprehensively investigate the influence of human genetic factors involved in the variability of susceptibility to viral infection and, in particular, in the variability of the clinical manifestations caused by SARS-CoV-2 [15, 22].

We embarked on the present systematic search of genetic association analyses in order to detect all possible hints suggesting the role of the host genome in determining the susceptibility to CoVs infections. We provide here a synthesis of evidence that could be helpful to design and conduct effective studies and, in turn, to find possible healthcare strategies aimed at facing the current COVID-19 pandemic.

## Methods

### Design and registration

The protocol was published in the PROSPERO repository of systematic reviews (registration number: CRD42020180860).

We designed a systematic review process to summarise the results and provide a field synopsis about the host genomic biomarkers associated with COVID-19 and other CoV-associated human syndromes caused by CoVs, and able to explain part of the variability in the relevant phenotype. Secondary key questions, if applicable, regarded the effect size of each associated genomic biomarker and the predictive value of such genomic biomarkers.

The protocol was developed according to the guidance of the PRISMA-P extension [23]. The PRISMA framework was applied to design reporting of results [24].

All records retrieved from databases were downloaded locally and managed by using the EndNote™ X8 software facilities. An ad hoc form for data abstraction and synthesis was defined on a worksheet.

### Eligibility criteria

Population: patients affected with COVID-19 and with other severe acute respiratory syndromes sustained by CoVs, with no restriction in a clinical setting. The COVID-19 phenotype was defined extensively to include infection, development of any clinical symptom or sign, grade of severity, specific organ involvement, sequelae or complications. Population studies on healthy individuals were not eligible.

Exposure: genomic biomarkers defined according to the European Medicine Agency definition (document EMEA/CHMP/ICH/437986/2006), that is “a measurable DNA and/or RNA characteristic that is an indicator of normal biologic processes, pathogenic processes, and/or response to therapeutic or other interventions”. This definition includes DNA variants such as single-nucleotide polymorphisms (SNPs), variability of short sequence repeats, haplotype, etc.

Outcomes: clinical diagnosis of SARS or associated syndromes; evidence of infection; atypical manifestation of illness; measurements of clinical outcomes (mortality, recurrence, severity, quality of life, etc.). Main outcome measure: odds ratio. Ancestry and sub-phenotypes were pre-defined as possible stratification parameters in data synthesis.

Study design*:* all study types showing effect size and significance were eligible (RCTs, cohort, case-control and cross-sectional studies).

Type of article: original peer-reviewed articles reporting quantitative results of the association between the CoVs-related phenotypes and any germline genomic variant.

Exclusion criteria*:* editorials and opinion papers were not considered. Pertinent narrative and systematic reviews were tagged in the screening phase and inspected for relevant references. Preprint articles that had not peer-reviewed were not eligible, though were considered for discussion.

### Strategy for literature search, data collection and data synthesis

The following databases were inspected: PubMed and Scopus for indexed published articles; medRxive and bioRxive for pre-print articles.

The search strategy was developed by two expert reviewers following an iterative process to ensure sensitivity. Two domains (“CoV” and “Genetics”) were defined combining MeSH terms and keywords using the Boolean operator “or”; the two domains were combined with “and”. Keywords for the dimension “genetics” were searched in the title field only. Several test runs showed that sensitivity was not affected, despite the remarkable reduction in the number of records retrieved [data not shown].

After interim results, a domain was added (“CoV-associated Biomarkers”) which targeted specific genes and genomic biomarkers found during the preliminary inclusion phase; the domain was combined with the “CoV” dimension with “and”; the resulting records were checked for duplicates and screened, then added to the final list of included articles. Search strings are reported in Supplementary Table S1.

Records were filtered for language (English) and date of publication (Jan 2003 to Apr 2020). The publication year field was restricted to the period from 2003 onwards, to include all articles published after the SARS outbreak dated 2003. The distribution of relevant publications indexed in PubMed confirmed a sharp rise since 2003 and a massive increase in 2020 (not shown).

Given the rapid evolution of the field, the literature search was replicated on June 17, in order to retrieve the latest articles and published versions of pre-print documents. To this purpose, PubMed and Scopus were interrogated.

Each identified study was indexed and manually abstracted. A total of 37 fields were pre-set for data extraction. Findings for all individual genetic variants under investigation were recorded, whether significantly associated or not. Some studies provided more than one data point (corresponding to one variant—one outcome measure entry) and were consequently represented with more than one record. The consistency of results between studies was analysed by gene and by the individual genomic biomarker. The study protocol did not envisage clinical recommendations after data synthesis.

Owing to the need for a rapid appraisal, the following modifications of the standard protocol for systematic reviews were applied, according to the current recommendations for rapid reviews [25, 26].

Grey literature was not systematically searched. Only articles in English were included in the screening phase and considered as eligible. One reviewer completed the screening phase according to an expedited PICO-based method previously described [27] and applying the rule of thumb “if in doubt keep it in” [24]. A second reviewer screened a sample of records. Eligible articles were inspected in-full by one reviewer and verified on title and abstract by a second independent reviewer. In case of doubts, the two reviewers discussed the accordance with the inclusion criteria. Data abstraction was drafted by one reviewer and verified independently by a second reviewer. Data synthesis was drafted by one reviewer and verified by all authors. Raw data were not requested. A formal assessment of the risk of bias was omitted. We relied on peer-review to ensure that included studies were methodologically sound, including proper and reliable statistical analysis (e.g. threshold for significance, correction for multiple testing, etc.). Therefore, unrefereed preprints were not included. It is noteworthy that most genetic association studies are based on cross-sectional or case-control design and as such are intrinsically affected by a high risk of bias.

## Results

### Systematic search

The systematic literature search in PubMed, Scopus, medRxive and bioRxive provided 1567 unique records and additional 362 records found in the last update (June 17, 2020). After the screening phase and full-text inspection, 32 articles fulfilled the criteria and were eligible for data abstraction. The detailed flow of literature assessment according to the PRISMA statement is depicted in Supplementary Figure 1.

Considering all variants tested as a predictor and the different outcome measures modelled as a dependent variable, >500 data points were included. In fact, this count should be further multiplied by the models tested in each study, i.e. allele and genotype association, dominant, recessive, codominant, and by the number of tests. However, we abstracted for each variant the statistics showing the larger effect size or the most significant result, if applicable.

### Populations

The 32 articles included for data abstraction analysed 26 independent cohorts—i.e. 3 cohorts were described in 4, 3 and 2 studies, respectively (Table 1).

**Table 1:** Articles included as eligible for data abstraction, ordered by year of publication and first author. For each study, main features, genes/loci examined and summary of the main findings are reported

Author, year	Population description	Country	Disease	Primary outcome	Other outcomes	Sample *N*	Cases *N*	Controls *N*	Notes on cohorts	Gene/locus	Conclusions	Ref.
Lin M, 2003	SARS cases admitted to Taipei Hospital, Taiwan	Taiwan	SARS	Manifest disease	Severity	134	33	101		HLA-A, HLA-B, HLA-DRB1	HLA-B*4601 nominally associated with severity (vs larger control group), not with infection after P correction	[28]
Chiu RWK, 2004	SARS patients admitted at Hong Kong Chinese University Hospital	Hong Kong	SARS	Manifest disease	Severity	496	168	328		*ACE2*	Negative results	[29]
Itoyama S, 2004	Vietnamese patients with SARS	Vietnam	SARS	Manifest disease	Hypoxemia	147	44	103	#	*ACE*	ACE nominally associated with severity	[30]
Ng MHL, 2004	SARS patients admitted at Hong Kong Chinese University Hospital	Hong Kong	SARS	Manifest disease	Severity	18864	90	18774		HLA-A, HLA-B, HLA-DR, HLA-DQ	HLA-B*0703 and -B*0301 nominally associated with the disease; no association with severity	[31]
Chan KC, 2005	SARS patients previously admitted at Hong Kong Hospitals	Hong Kong	SARS	Manifest disease	Severity	466	140	326		*ACE*	Negative results	[32]
Hamano E, 2005	SARS patients	Vietnam	SARS	Manifest disease	Severity (oxygen therapy)	147	44	103		*OAS-1, MxA, PKR*	Nominal association of OAS-1 with disease but not severity; nominal association of MxA with severity only	[33]
Ip WK, 2005	SARS patients previously admitted at 5 Hong Kong Hospitals	Hong Kong	SARS	Manifest disease	Serum MBL level; severity (death)	1757	569	1188		*MBL*	Association of -221 Y allele with disease and MBL level; no association with mortality	[34]
Itoyama S, 2005	Vietnamese patients with SARS	Vietnam	SARS	Manifest disease		147	44	103	#	*ACE2*	Negative results	[35]
Yuan FF, 2005	SARS patients admitted at Prince of Wales Hospital, Hong Kong	Hong Kong	SARS	Manifest disease	Severity (ICU or death)	380	180	200	§	*FcγRIIA, MBL*	No association with MBL; FcγRIIA nominally associated with severity	[36]
Zhang H, 2005	SARS patients from Bejing, China	China	SARS	Manifest disease	Serum MBL level	744	352	392		*MBL*	Association of nt 54 B allele with disease and MBL level	[37]
Chan VS, 2006	SARS patients admitted at four hospitals in Hong Kong	Hong Kong	SARS	Manifest disease		1127	285	842	$	*CLEC4M (L-SIGN)*	CLEC4M nominally associated with homozigosity (protective). Functional analysis consistent with genetic risk.	[38]
Chong WP, 2006	SARS cases retrospecivelly selected in Hong Kong and Bejing, China	Hong Kong	SARS	Manifest disease	Severity (death)	925	476	449		*IFN-γ, IL-10, TNF-α*	No association with IL-10 and TNFalfa; IFNγ nominally associated with disease, not associated with severity	[39]
He J, 2006	SARS patients from Bejing, China	China	SARS	Manifest disease		66	66	64	Cohort assessed for risk factors	*OAS-1, MxA*	Nominal association of OAS-1 and MxA with disease. For both variants association found in dominant model only.	[40]
Chen WJ, 2006	SARS cases retrospecivelly selected from the National Taiwan database	Taiwan	SARS	Positive NPS	Manifest disease	188	94	94		*ACE2, ACP5, ADAR, AIP, ANPEP, B2M, CAT, CCL5/RANTES, CD209, CIITA, CXCL9, CXCL10, CYP17A1, EIF2AK3, EIF2S1, EIF4G1, ESR1, FGL2, FN1, G6PD, GNB3, GPX1, GSS, HMOX1, IFNAR1, IFNAR2, IFNG, IFNGR1, IFNGR2, IL1A, IL1B, IL1RN, IL4, IL6, IL10, IL10RB, IL12A, IL15, IL18, IRF1, IRF3, IRF7, MBL2, MX1, NFRKB, OAS1, PRDX2, PRKRA, PTGS2, RelB, RFX5, RNASEL, SERPINB3, SH2DIA, SLAMF1, SOCS1, SOCS3, SOD1, TBF, TFRC, TGFB1, TLR3, TLR4, TRAF6, WSX1*	Nominal association of detectable NPS with 4 genes (IL1A, IL18, FGL2, RelB) in the patients’ cohort; no association with infection in the case-control study	[41]
Chen YM, 2006	Employees of the Municipal Hoping Hospital, Taipei	Taiwan	SARS	Seropositivity		100	20	80		*HLA-A, HLA-B, HLA-Cw, HLA-DQB1, HLA-DRB1*	HLA-Cw0801 nominally associated with SARS infection	[42]
Chan KYK, 2007	SARS patients previously admitted at 6 Hong Kong Hospitals	Hong Kong	SARS	Manifest disease	LDH level, WBC	1723	817	906	$	*ICAM3, FCER2, CD209 (DC-SIGN), CLEC4M (L-SIGN)*	ICAM3 possibly associated with severity, via LDH level as a proxy	[43]
Ng MW, 2007	SARS cases retrospectively selected in Hong Kong and Bejing, China	Hong Kong	SARS	Manifest disease	Severity (ICU or death)	1073	495	578		*CCL5/RANTES, IP-10, Mig*	No association with IL-10 and Mig; *CCL5/RANTES* -28C > G is associated with disease (discovery cohort only) and severity (both discovery and validation cohorts). Association between CCL5/RANTES and severity was validated in an independent cohort of Chinese patients	[44]
Yuan FF, 2007	SARS patients admitted at Prince of Wales Hospital, Hong Kong	Hong Kong	SARS	Manifest disease	Severity (ICU)	350	152	198	§	*TLR2, TLR4, CD14*	No informative variant in *TLR2* and *TLR4*; *CD14* nominally associated with severity	[45]
Li H, 2008	SARS patients retrospectively collected	Hong Kong	SARS	Manifest disease	Severity (ICU or death)	353	181	172		*FCER2, CLEC4G, CD209, CLEC4M (L-SIGN)*	Negative results (4 genes in the C-type lectin genes cluster, chr. 19).	[46]
Wang S, 2008	SARS patients admitted at Tianjin Hospital, China	China	SARS	Manifest disease	Interstitial lung fibrosis; femoral head necrosis	208	75	133		*TNF-α*	No association with infection and interstitial lung fibrosis; nominal association with femoral head necrosis	[47]
Xiong P, 2008	SARS patients from Guangdong province, China	China	SARS	Manifest disease	Severity	498	95	403		HLA-A, HLA-B, HLA-DRB1	Negative results	[48]
Keicho N, 2009	Vietnamese patients with SARS	Vietnam	SARS	Manifest disease	Seropositivity	145	44	101	#	HLA-A, HLA-B, HLA-C, HLA-DRB1, HLA-DQB1	HLA-DRB1*12 possibly associated with disease	[49]
Wang Y, 2009	SARS patients from Bejing and Guangzhou, China	China	SARS	Manifest disease		899	376	523		*MASP2*	Negative results (MASP is a downstream protein of MBL).	[50]
Chan KYK, 2010	SARS patients previously admitted at Hong Kong Hospitals	Hong Kong	SARS	LDH level		681	681	n/a	$	*CD209 (DC-SIGN); combined CD209 and ICAM3*	*CD209* and *CD209* + *ICAM3* possibly associated with severity, via LDH level as proxy	[51]
Ching JCY, 2010	SARS patients previously admitted at 6 Hong Kong Hospitals	Hong Kong	SARS	Manifest disease	Severity (assisted ventilation and other not reported measures)	1210	792	418	$	*MxA*	Nominal association of MxA with disease, not with severity; *OAS*-*1* + *MxA* dyplotype also analysed	[52]
Ng MH, 2010	SARS patients recruited from the Hong Kong Department of Health database	Hong Kong	SARS	Manifest disease		210	102	108	Controls were contact of patients; families also enrolled	HLA-A, HLA-B, HLA-Cw, HLA-DQ, HLA-DR	Negative results: nominal association with disease (increase in DRB4*01010101 frequency, decrease in HLA-B*1502 and HLA-DRB3*030101 frequency in patients) not surviving to correction. Replication of previous study failed. Analysis of family data was done.	[53]
Wang SF, 2011	Affected health care workers followed-up at the Dept. of Health, Taipei	Taiwan	SARS	Manifest disease	Prolonged neutralising antibody expression	97	56	41		HLA-B, HLA-Cw, HLA-DR	HLA-Cw1502 and DR0301 possibly associated with resistance to SARS infection	[54]
Yuan FF, 2014	SARS patients admitted at Hong Kong Chinese University Hospital	Hong Kong	SARS	Manifest disease	Severity	18950	176	18774	48 cases classified as severe	HLA-A, HLA-B, HLA-DR	Negative results	[55]
Hajeer AH, 2016	Patients with laboratory-confirmed MERS-CoV infection admitted at King Abdulaziz Medical City, Riyadh	Saudi Arabia	MERS	Manifest disease	Severity	184	23	161		HLA-DRB1, HLA-DQB1	HLA-DRB1*11:01 nominally associated with severe MERS	[56]
Kuo CL, 2020	COVID-19 patients from the UN Biobank		COVID-19	COVID-19 positivity status		323570	622	322948	General population as control	*APOE*	*APOE* ε4 homozygous genotype associated, independent of preexisting dementia	[57]
Wang W, 2020	Donors of convalescent plasma who recovered from COVID-19 in Zhejiang, China	China	COVID-19	Manifest disease		3630	82	3548	No severe case; 242 controls for HLA-DP1 locus	HLA-A, HLA-B, HLA-C, HLA-DQ, HLA-DR, HLA-DP	HLA haplotype C*07:29 and B*15:27 nominally associated with SARS-CoV-2 infection	[58]
Zhang Y, 2020	Patients with COVID-19 admitted to Beijing Youan Hospital, China	China	COVID-19	Disease severity		80	80		24 severe cases vs 56 mild	*IFITM3*	Association with severity	[59]

Notes and legend: gene and polymorphism names were reported as described in articles; the dbSNP nomenclature was added in brackets; statistics was reported if the significant association was found, not significant was annotated otherwise.

*ICU* intensive care unit, *LDH* lactate dehydrogenase, *WBC* white blood counts, *LR* logistic regression, *ns* not significant, *n/r* not reported, *n/a* not applicable, #: Same cohort used by Itoyama S 2004, 2005, Keicho N 2009; § Same cohort used by Yuan FF 2005, 2007; $: Same cohort used by Chan VS 2006, Chan KYK 2007, 2010, Ching JCY 2010.

Consistently with the outbreaks experienced in the last decades and the period considered for inclusion, SARS infection was by far the most frequent endpoint. Therefore, all studies investigated patients of Asian ancestry—Chinese Han and Vietnamese—except the only study which focused on MERS in Saudi Arabia and one recent analysis on individuals of European ancestry based in the UK Biobank.

Patient series were ascertained according to the current diagnostic criteria and recruited retrospectively. No randomisation procedure was found in eligible studies. The healthcare setting—i.e. whether in-patient or out-patient clinic, follow-up, etc.—was seldom reported.

The control series resulted to be consistent for ancestry. No paired case-control enrolment was applied.The discovery samples were heterogeneous in size across studies, ranging from 44 to 323,570, while the patient series count was from 20 to 817 (Table 1).

### Outcomes

Out of the 32 studies, 27 considered the disease status as the primary outcome measure, 3 seropositivity or positive nasopharyngeal shedding, 1 used disease-associated biomarkers (serum LDH level, white-blood count) and 1 measured COVID-19 severity (Table 1). A large proportion of association studies considered the severity of the disease course as a secondary outcome, as measured by admittance to intensive care unit, administration of oxygen therapy, or death. The clinical variables analysed as secondary outcomes were ascertained on a subset of the patient series and modelled as binary variables (Table 1).

### Designs

All studies implemented a case-control design. If the association with clinical outcome was tested, a subset of patients was analysed and classified in a binary variable. Genotype and allele frequencies were compared by using univariate parametric statistics. Several studies applied also multivariate statistics including age and sex as covariate; one considered lifestyle-related risk factors; one corrected for putatively associated variants.

All studies assumed 0.05 as the threshold of significance (as reported or inferred from article reading). Correction for multiple testing was not common in included studies, though hypothesis testing in genetic association studies implies multiple testing (see Table 3). One attempt to validate findings on an independent cohort was found [44].

### Genetic association

Table 1 reports all studies included. For each study, we reported genes/loci examined and a summary of main findings. Twenty-two articles reported the association analysis of CoV-related phenotypes with single-gene variants, either in coding or regulatory region. Ten genes were analysed in more than one study (Table 2), namely: *ACE2*, *CLEC4M* (L-SIGN), *MBL*, *MxA* (3 studies); *ACE*, *CD209*, *FCER2*, *OAS-1*, *TLR4*, *TNF-α* (2 studies). Out of these, only *MBL* and *MxA* provided positive signal of association in at least two studies. The *CCL5*/*RANTES* gene was reported in one article (Table 1) which investigated also an independent cohort of the same ancestry (i.e. Chinese) as a replication study; the second cohort provided a significant signal of association with severity but not with the manifest disease [44] (Table 3).

**Table 2:** Genes/loci examined in included studies, ordered by the number of relevant studies. Genes and HLA loci were listed separately

*N* of studies	Gene
3	*ACE2*
3	*CLEC4M (L-SIGN)*
3	*MBL*
3	*MxA*
2	*ACE*
2	*CD209*
2	*FCER2*
2	*OAS-1*
2	*TLR4*
2	*TNF-α*
1	*ACP5, ADAR, AIP, ANPEP, B2M, CAT, CCL5, CD14, CIITA, CLEC4G, CXCL10, CXCL9, CYP17A1, EIF2AK3, EIF2S1, EIF4G1, ESR1, FcγRIIA, FGL2, FN1, G6PD, GNB3, GPX1, GSS, HMOX1, IFNAR1, IFNAR2, IFNG, IFNGR1, IFNGR2, IL10, IL10RB, IL12A, IL15, IL18, IL1A, IL1B, IL1RN, IL4, IL6, IP-10, IRF1, IRF3, IRF7, MASP2, MBL2, Mig, MX1, NFRKB, OAS1, PRDX2, PRKRA, PTGS2, RANTES, RelB, RFX5, RNASEL, SERPINB3, SH2DIA, SLAMF1, SOCS1, SOCS3, SOD1, TBF, TFRC, TGFB1, TLR2, TLR3, TRAF6, WSX1*
	**HLA haplotype**
10	HLA-DR
9	HLA-B
8	HLA-A
5	HLA-Cw
5	HLA-DQ

**Table 3:** Significant data points ordered by gene and by variant, according to the positive signals of association found across studies (not significant data points were omitted). Sample size and effect size were reported as abstracted from the source article. Genes and HLA loci were listed separately

Author, year	Disease	Ancestry	Outcome	Sample *N*	Cases *N*	Controls *N*	Notes on sample	Gene/locus	Variant	Risk allele	Effect size, OR	95% CI	*P* value			Notes on statistics	Main finding	Ref.
Itoyama S, 2004	SARS	VIE	Hypoxemia	44	44	n/a	#	*ACE*	I/D (rs4646994)	D	3.04	1.12–8.25	0.029			22 severe vs 22 non-severe cases	No association with disease	[30]
Kuo CL, 2020	COVID-19	EUR	COVID-19 positivity status	323570	622	322948	General population as control	*APOE*	rs429358 + rs7412	ε4	2.31	1.71–3.35	<0.001		m	Statistics from the whole cohort; strata available	Association with the ε4 homozygous genotype	[57]
Ng MW, 2007	SARS	CHI	Manifest disease	1073	495	578		*CCL5*	rs2107538	G	2.8	2.11–3.71	<0001	c	m	Allele-associated OR reported	Association with disease	[44]
Ng MW, 2007	SARS	CHI	Severity (death)	495	495	n/a		*CCL5*	rs2107538	G	2.1	1.30–3.39	0.002	c	m	57 deaths vs438 survivals; allele-associated OR reported	Association with death (57 deceased vs 438 survived patients)	[44]
Ng MW, 2007	SARS	CHI	Severity (ICU)	356	356	n/a		*CCL5*	rs2107538	G	2.78	1.37–5.63	0.005	c	m	20 severe cases vs 336; allele-associated OR reported	Association with severity	[44]
Yuan FF, 2007	SARS	CHI	Manifest disease	152	152	n/a	§	*CD14*	159C/T (rs2569190)	C	n/r	n/r	0.027			26 severe cases vs 136 mild	*CD14* associated with severity	[45]
Chan KYK, 2010	SARS	CHI	LDH level	681	681	n/a	$	*CD209 (DC-SIGN)*	-366AG, promoter region	G	0.4	0.19–0.85	0.014			LDH modelled as binary variable; univariate statistics reported	Association with lower LDH level	[51]
Chan VS, 2006	SARS	CHI	Manifest disease	1127	285	842	$	*CLEC4M (L-SIGN)*	exon 4 tandem repeat	homozigosity	0.691	0.523–0.913	0.009			.	Homozigosity associated with resistance to infection	[38]
Chan KYK, 2010	SARS	CHI	LDH level	677	677	n/a	$	*combined CD209 and ICAM3*	-366AG, promoter region + rs2304237	A; A	4.34	1.34–14.12	0.021			LDH level modelled as binary variable; univariate statistics reported	Association with higher LDH level	[51]
Yuan FF, 2005	SARS	CHI	Severity (ICU or death)	380	26	200	§	*FcγRIIA*	R131H (rs1801274)	R	3.2	1.1–9.1	0.03			26 patients vs controls	Nominal association with severity	[36]
Chen WJ, 2006	SARS	CHI	Positive NPS	188	94	94	0	*FGL2*	+158 (rs2075761)	A	4	1.39–11.48	0.031			GA genotype; outcome modelled as binary variable	Nominal association of NPS with 4 genes (*IL1A*, *IL18*, *FGL2*, *RelB*)	[41]
Chan KYK, 2007	SARS	CHI	LDH level	677	677	n/a	$	*ICAM3*	rs2304237	C (Gly)	4.31	1.37–13.56	0.0067			LDH modelled as binary variable; P corrected for 2 tests	ICAM3 associated with LDH level	[43]
Zhang Y, 2020	COVID-19	CHI	Disease severity	80	80	n/a	24 severe vs 56 mild	*IFITM3*	rs12252	C	6.37	n/r	<0.001			CC vs other genotypes; CI n/r	Association with severity	[59]
Chong WP, 2006	SARS	CHI	Manifest disease	925	476	449		*IFN-γ*	rs2430561	A	5.19	2.78–9.68	<0.001		m	Recessive model; multivariate LR n/r	Association with one SNP	[39]
Chen WJ, 2006	SARS	CHI	Positive NPS	188	94	94		*IL18*	-607 (rs1946518)	T	10.6	2.03–55.0	0.014			Outcome modelled as binary variable	Nominal association of NPS with 4 genes (*IL1A*, *IL18*, *FGL2*, *RelB*)	[41]
Chen WJ, 2006	SARS	CHI	Positive NPS	188	94	94		*IL1A*	−889 (rs1800587)	T	10.2	1.82–56.8	0.008			Outcome modelled as binary variable	Nominal association of NPS with 4 genes (*IL1A*, *IL18*, *FGL2*, *RelB*)	[41]
Ip WK, 2005	SARS	CHI	Manifest disease	1541	353	1188		*MBL*	−221X/Y (rs7096206); ex1 54A/B (rs1800450)	YB	1.55	1.21–1.99	<0.001		m	Statistics n/r	Association of YB haplotype with disease	[34]
Ip WK, 2005	SARS	CHI	Serum MBL level	353	353	n/a		*MBL*	−221X/Y (rs7096206); ex1 54A/B (rs1800450)	YB	n/r	n/r	n/r		m	Statistics n/r	Association of YB haplotype with low serum MBL level	[34]
Zhang H, 2005	SARS	CHI	Manifest disease	744	352	392		*MBL*	230[codon 54]A/B (rs1800450)	B	1.73	1.25–2.39	0.00086	c			Association of B allele with disease	[37]
Zhang H, 2005	SARS	CHI	Serum MBL level	744	352	392		*MBL*	230[codon 54]A/B (rs1800450)	B	1.67	1.21–2.29	0.00187	c			Association of B allele with MBL level	[37]
Hamano E, 2005	SARS	VIE	Severity (oxygen therapy)	44	44	n/a		*MxA*	rs2071430 − 88)	G	3.75	1.08–10.7	0.0346				Association with severity	[33]
He J, 2006	SARS	CHI	Manifest disease	66	66	64	Cohort assessed for risk factors	*MxA*	rs2071430	T	3.22	1.13–9.18	0.029		m		Association with AG genotype, dominant G protective	[40]
Ching JCY, 2010	SARS	CHI	Manifest disease	1210	792	418	$	*MxA*	rs17000900 (−123)	A	0.58	0.41–0.82	0.002	c	m		Association with resistance to infection	[52]
Hamano E, 2005	SARS	VIE	Severity (oxygen therapy)	44	44	n/a	0	*OAS-1*	rs3741981	G	2.68	1.17–6.15	0.0178				Association with disease	[33]
He J, 2006	SARS	CHI	Manifest disease	66	66	64	Cohort assessed for risk factors	*OAS-1*	rs2660	G	0.38	0.14–0.98	0.047		m		Association with GT genotype, dominant T	[40]
Chen WJ, 2006	SARS	CHI	Positive NPS	188	94	94		*RelB*	+23692 (rs2288918)	T	7.2	1.47–35.3	0.034				Nominal association of NPS with 4 genes (*IL1A*, *IL18*, *FGL2*, *RelB*)	[41]
Wang S, 2008	SARS	CHI	Manifest disease	54	54	n/a		*TNF-α*	−1031	C	6	1.60–22.55	0.04			24 severe cases vs 30 mild	Nominal association with femoral head necrosis	[47]
Wang S, 2008	SARS	CHI	Manifest disease	54	54	n/a		*TNF-α*	−863	A	8.4	1.76–40.02	0.01			24 severe cases vs 30 mild	Nominal association with femoral head necrosis	[47]
Chen YM, 2006	SARS	CHI	Seropositivity	100	20	80		HLA-Cw,		Cw0801	3.4	1.50–7.58	0.003	c		Number of tests not shown	Association found in recessive model	[42]
Lin M, 2003	SARS	n/r	Manifest disease	134	33	101		HLA-B		B*4601	10.62	2.80–40.26	0.0279	c		6 severe cases vs 101 health care workers	Association with both infection and severity	[28]
Ng MHL, 2004	SARS	CHI	Manifest disease	18864	90	18774		HLA-B		B*0301	0.06	0.01–0.47	0.0042	c		.	Association with resistance to infection	[31]
Ng MHL, 2004	SARS	CHI	Manifest disease	18864	90	18774		HLA-B		B*0703	4.08	2.03–8.18	0.0022	c		.	Association with infection	[31]
Wang W, 2020	COVID-19	CHI	Manifest disease	3630	82	3548		HLA-B		B*15:27	3.59	1.72–7.50	0.03	c			Association with infection	[58]
Wang W, 2020	COVID-19	CHI	Manifest disease	3630	82	3548		HLA-C		C*07:29	130.2	5.28–3211	0.025	c			Association with infection	[58]
Wang SF, 2011	SARS	n/r	Manifest disease	97	56	41		HLA-Cw		Cw 1502	0.17	0.04–0.81	0.01			.	Association with resistance to infection	[54]
Hajeer AH, 2016	MERS	SAU	Manifest disease	184	23	161		HLA-DRB1		DRB1*11:01	6.11	1.36–24.76	0.022	c		.	Association with the disease	[56]

Notes and legend: gene and polymorphism names were reported as described in articles; the dbSNP nomenclature was added in brackets.

*CHI* Chinese, *EUR* European, *SAU* Saudi Arabia, *VIE* Vietnamese, *ICU* intensive care unit, *LDH* lactate dehydrogenase, *WBC* white blood counts, *NPS* nasopharyngeal shedding

#: Same cohort used by Itoyama S 2004, 2005, Keicho N 2009; § Same cohort used by Yuan FF 2005, 2007; $: Same cohort used by Chan VS 2006, Chan KYK 2007, 2010, Ching JCY 2010; *LR* logistic regression, *ns*: not significant, *n/r* not reported, *n/a* not applicable, *c* corrected for multiple testing, *m* multivariate analysis

Ten studies investigated the hypothesis of association with HLA loci (Tables 1 and 2). Three of these reported negative results: two independent studies analysed Chinese patients with SARS [48, 55]; one study [53] failed to replicate the association with HLA-B*0703 and HLA-DRB1*0301 previously reported [31].

Positive signals were found in 7 studies (5 on SARS, 1 in MERS and COVID-19). The following HLA haplotypes were found differentially distributed in patients with respect to the reference population: B*4601; B*0703; B*0301; Cw0801: DRB1*1202; Cw1502; DR0301; DRB1*11:01; C*07:29; B*15:27. None of these provided positive signals in more than one cohort (Table 3).

## Discussion

This is the first comprehensive review which systematically collected all studies examining the involvement of human genetic variants in severe CoV infections, including COVID-19. Patarčić and co-workers had published the first extensive review including all studies investigating five common respiratory tract infectious diseases—pneumonia, tuberculosis, influenza, respiratory syncytial virus and SARS-CoV. At that time (last update: August 2015), they could cover the SARS-CoV-1 outbreak, though with some limitations such as the exclusion of HLA haplotypes [14]. A narrative review published well before the first SARS outbreak had pointed to CoV infection with a focus on animal studies [60].

By developing this comprehensive review, we recapitulated the body of knowledge about the influence of host germline genetic variants on the clinical phenotypes associated with CoVs infection. In total, we examined 32 articles that met the criteria and were eligible for data extraction.

HLA haplotypes were examined in ten studies spanning the entire period from the SARS-CoV-1 outbreak in 2003 up to the SARS-CoV-2 pandemic in 2020. The remaining 22 studies tested the hypothesis of association with single-gene variants. A summary of the findings is reported below.

### Association signals from single-gene studies

Two different studies reported an association between mannose-binding lectin (MBL) polymorphisms and susceptibility to SARS-CoV infection [34, 37]. MBL is a serum protein of the collectin family and plays a critical role in the innate immune response. MBL binds, by its multiple carbohydrate recognition domains, to repeating mannose and *N*-acetylglucosamine sugar motifs frequently present on the microbial surfaces of bacteria, viruses and protozoa [61]. After binding to a pathogen, MBL activates the complement system via MBL-associated serine protease or promotes phagocytosis by acting as an opsonin [62]. Polymorphisms in the promoter and coding regions of the *MBL* gene seem to have functional effects on MBL serum levels. The variant alleles causing low plasma concentrations of functional MBL were described as associated with an increased risk of developing infections [63]. Since both included studies the variants associated with lower MBL levels were observed more frequently in patients with SARS, it might be speculated that MBL is able to bind the spike protein of SARS-CoV and contributes to the defence of the host cell.

Abnormalities in the production levels of several cytokines such as IL-1, IL-2, IL-4, IL-6, IL-8, IL-10, IFN-γ, TNF-α and TGF -β1 were detected in patients with SARS-CoV infection [64]. Cytokines are key mediators of the inflammatory response and are important for host defence against a wide variety of viruses, by participating in the regulation of both innate immunity and inflammatory processes. The individual level of cytokines is extremely variable with an important contribution of the genetic component since it was demonstrated that polymorphisms located in genes coding for cytokines can influence their transcriptional activity [65]. For these reasons, the genetic variants of inflammatory cytokines genes were extensively studied in patients with CoVs infection. However, we found one study reporting a significant association with SARS susceptibility, namely the minor allele of rs2430561 polymorphism in *IFN-γ* gene in a Chinese cohort [39]. Moreover, variants in *Il1A* and *IL18* showed nominal association with nasopharyngeal shedding [41], and variants in the *TNF-α* promoter region were found as associated with femoral head necrosis [47]. None of these findings was replicated to date.

Type 1 interferon (IFN-a/b) plays an important role in the innate immune response against viral infections. Among the antiviral proteins induced by IFN1, 2'-5' oligoadenylate synthetase 1 (*OAS1*) and myxovirus resistance 1 (*MxA*) harbour genetic variants that might affect susceptibility to the SARS-CoV infection and progression [66]. OAS1 can bind double-stranded RNA and activate a latent ribonuclease, RNase L, which cleaves cellular and viral RNAs. Associations between SARS and *OAS1* genetic variants located in exon 3, exon 6 [33] and in region 3’ UTR [40] were found in our study. In particular, the rs1131454 polymorphism in exon 3, located near the dsRNA binding domain, causes the aminoacid substitution Gly162Ser and could affect its activity [33]. MxA is a cytoplasmic protein that belongs to the dynamin family and shows activity against several viruses. The variant alleles of rs2071430 and rs17000900 SNPs, located in the promoter region, were shown to be associated with increased promoter activity and could influence the binding affinity with nuclear protein [67]. However, the precise mechanism of antiviral action of MxA was not elucidated so far. The genetic association studies that were found eligible in this study are discordant. In fact, while two studies reported that the minor allele of rs2071430 SNP is associated with SARS-CoV [40] and hypoxia caused by the infection [33], Ching at al. claim that this allele confers resistance to the virus [52]. Yet, the formers were conducted on small cohorts and found positive signal with different outcomes. Despite the promising background, the association of *MxA* with CoVs-related phenotypes needs to be investigated in large cohorts by applying a robust study design.

One article reported the association between SARS susceptibility and a genetic variant in the *RANTES* (Regulated upon Activation, Normal T cell-Expressed and Secreted) gene, also known as *CCL5* [44]. *RANTES* codes for a chemokine responsible for the recruitment of eosinophils, lymphocytes, monocytes and basophils at the site of inflammation [68]. The study showed that the minor allele -28G is associated with severe clinical outcome in SARS Chinese patients, and increases *RANTES* expression and enhances NF-κB binding in vitro, thus suggesting its role in promoting inflammation [44]. This was the only study included in the eligible series which provided a functional hint linking the association signal with a putative biological relevance—yet, the promising findings warrant independent replications.

After the spread of the COVID-19 pandemic, it has been speculated on the possible role of *ACE2* variants, as well as other factors implicated along the pathway of SARS-CoV2 infection. In fact, biochemical interaction studies and crystal structure analyses revealed that the SARS-CoV-2 spike protein has a strong binding affinity with the human ACE2 extracellular domain [16, 17, 69, 70], and that SARS-CoV-2 and the original SARS-CoV display a high degree of homology [16, 71, 72], demonstrating that both viruses recognise the ACE2 peptidase domain as a receptor. Several studies compared the allele frequency of relevant *ACE2* polymorphisms obtained from reference data with the prevalence of COVID-19 in different countries [19, 73, 74]. These dry lab investigations produced interesting hypotheses. However, the articles did not satisfy our inclusion criteria as the analyses did not entail patients’ genetic data. Although the authors correctly pointed to possible confounders and adjusted the statistical models through a multivariate approach, these studies assumed that (i) patients affected with COVID-19 were a random sample of the reference population, and (ii) patients did not differ in any determinant of either disease or severity, other than the polymorphisms under investigation—two assumptions that cannot be postulated.

Although the role of the host immune system in the development and clinical course of the severe respiratory syndrome caused by SARS-CoV1 and SARS-CoV-2 has been increasingly apparent, it is also worth to underline that some promising candidate genes, namely *IL4* and *IL6*, were not sufficiently investigated so far. One out of the 32 eligible studies included variants of both genes in a panel of 65 loci, with negative results [41].

In the systematic review and meta-analysis of studies addressing host genetic factors implicated in five common respiratory tract infectious diseases—i.e tuberculosis, influenza, respiratory syncytial virus (RSV) and SARS-CoV—published in 2015 [14], Patarčić and co-workers reported *IL4* as the single result that was significant in pooled analyses, and marginally significant in disease-specific meta-analyses. *IL4* promotes both T-cell and B-cell differentiation and provides a balance between Th1 and Th2 response [75]. Given its pivotal role in shaping the nature of the immune response, it is conceivable that even subtle modifications of IL4 function and expression may substantially affect the immune response and influence the course of CoVs infection.

IL-6 is one of the pro-inflammatory cytokines whose levels appear to increase sharply in patients with COVID19 [76]. In particular, the highest circulating levels of IL-6 were observed in patients with respiratory dysfunction, suggesting that SARS-CoV-2 infection triggers a cytokine-mediated lung damage mechanism [77]. Recent meta-analyses confirmed that elevated IL-6 levels are closely associated with the severity of COVID-19 [78–80], further suggesting that *IL6* genetic variants should be considered as a potential determinant of the host response against CoVs [81]. Based on these data, COVID19 patients with pneumonia were also treated with drugs directed against IL-6 receptor (IL6R), such as tocilizumab, with promising results [82]. The association of *IL6R* gene polymorphisms with the effectiveness of tocilizumab was found in rheumatoid arthritis [83] and was recently investigated in patients affected with COVID-19 [84]. Conversely, our systematic search did not find any genetic association analysis targeting *IL6R* in CoV-related diseases.

We did not focus on pharmacogenetics of treatments against CoV infections, as a response to drugs is a distinct phenotype which deserves a specific focus. Preliminary data pointed to germline genetic variants influencing the efficacy of specific therapies administered in patients with COVID-19 [84]. This line of research may disclose basic mechanisms linked to the disease pathophysiology and provide additional hints on the genetic determinants of the host response [85, 86].

### Association signals from HLA haplotypes

As the involvement of immune response in the clinical course of CoV-related diseases had been apparent since the first severe cases with SARS occurred in 2003, several efforts have been made to identify susceptibility or resistance factors associated with the variability of HLA alleles in the human population. This review included ten studies that investigated the hypothesis of association with HLA. In summary, no HLA haplotype was found to be significantly associated with CoV-related phenotypes in more than one study. Moreover, most studies appear rather weak in terms of study design and robustness of statistics (see also below on the methodological issues).

Our findings are in line with the recent review by Sanchez-Mazas [87], who critically resumed both case-control studies and structure analyses (bioinformatics and in vitro), concluding that rigorous case-control studies should be combined with experimental HLA binding and T cell response assays in order to elucidate the role of the huge HLA variability in response to CoVs.

### Methodological issues

In general, the sample size of eligible studies was limited by the absolute number of cases available for clinical and genetic ascertainment, and supposedly by the logistic constraints of an outbreak. A few studies, however, used a very small case-control sample—i.e. <100 cases, see Tables 1 and 3—thus implying a very high prior risk of both type I and type II errors. The limited sample size was prevented from applying an unbiased, agnostic approach, such as genome-wide association analyses. In fact, all studies considered in the present review were based on the candidate gene approach, sensibly due to the limited sample size. As long as the candidate approach is used, a relative increase in the prior likelihood of association could reduce the risk of type I error. To this aim, the latest studies on SARS-CoV-2 infection could inform on the biological relevance of gene products. Gene ontology, in turn, may allow to extend the focus from a single gene to a network of genes related to the same pathway. To accomplish this approach, statistical modelling and analyses should be more sophisticated with respect to the simple statistics applied in most genetic association studies. The use of polygenic models might overcome the intrinsic constraint due to low effect size attributable to single-gene variants and allows to explain part of the missing heritability in complex disorders. This is the case, for instance, of Alzheimer’s disease, for which complex trait analysis has been proved to successfully interpret a large proportion of the genetic variance [88], as well as breast cancer, in which multifactorial models incorporating multiple genetic variants are under validation [89, 90]. By the way, family studies should be warranted to estimate the heritability of the COVID-19 phenotypes through a formal assessment and provide the grounded basis for subsequent hypotheses.

Many nominally significant signals of association listed in tables would not survive to rigorous corrections for multiple testing (see Table 3). Unfortunately, the actual set of data is not suitable for post hoc corrections, as the number of tests was not declared, and statistical models were not reported in most instances.

A few attempts to replicate and validate findings were found. Ng MW and co-workers [44] found a positive association signal of rs2107538 in the *RANTES*/*CCL5* gene with manifest disease and severity of the clinical course. The analysis on an independent cohort succeeded in replicating only the association with severity as measured by admission to the intensive care unit, by using a sample of 20 severe vs 336 mild patients [44]. The original findings [31] were matched in an independent study which applied a conservative correction for multiple testing, failing to replicate the association of SARS with HLA-B*0703 and HLA-DRB1*0301 [53].

Study design is another limitation of all investigations evaluated in this systematic search. Patient and control series were retrospectively collected based on the clinical diagnosis with no definite procedure for enrolment, thus inflating the risk of selection bias. Most studies used the general population as control, with no matched case-control design. Control series were matched for ancestry but not paired with patients for other relevant variables. Most important, in the majority of studies the exposure to CoV had not been established—therefore, control individuals could be defined as “non-cases”, but their susceptibility to the disease (i.e. the phenotype under study) had not been ascertained.

Studies on HLA used reference data from the general population as the control series (see Table 3).Reference HLA data provide the best estimates of haplotype frequencies for a given ancestry and geographical area. However, these data sets do not convey information on the phenotype under study, namely the susceptibility to CoV infection and the associated illness. Whether these data set can be assumed as phenotype-negative control population is arguable.

Individuals who acquired the infection and developed a severe form of COVID-19 can be presumed to lie in the extreme of the curve corresponding to the most susceptible individuals. On the opposite extreme, there might be located persons sharing the basic risk factors (such as spouses) who were ascertained as either seronegative or mildly affected. The latter individuals could be reasonably classified as resistant—either to infection or severe manifestations—and could serve as matched controls with respect to selected cases. Our recognition found one study that implemented a procedure to enrol control individuals [53].

To deploy this approach, clinical registries and linked biobanks are a helpful source of information on COVID-19 phenotype variability. The UK Biobank (UKB) has already provided pieces of evidence on COVID-19 [91], and one study matched the inclusion criteria of the present review. By interrogating the UKB, Kuo and co-workers found that *ApoE* ε4 homozygous individuals are more likely to be COVID-19 test positives compared with ε3 homozygotes, independently from dementia [57].

### Limitations of the study

This systematic review and summary of findings should be weighted for some limitations.

The rapid protocol may have limited the sensitivity of the literature search; thus, some relevant articles might have been missed. To reduce this risk, we performed an extensive hand-search of reference lists and updated the search in order to include the latest studies and unrefereed articles previously found.

The present study also suffers from the inability to adjust significance and effect size for multiple testing and basic covariates, such as age, sex and comorbidity that were found to account for most of the variability in the clinical manifestations of CoV-associated diseases, particularly COVID-19 [10, 11]. The paucity of such information is an intrinsic limit of the examined literature. In fact, the lack of multiple data sets for each genetic risk factor, as well as the heterogeneity of samples and outcome measures, has prevented any post hoc analyses. As a consequence, publication bias towards nominally significant results may be supposed but could not be estimated. The short term of our rapid protocol implied that raw data and unpublished data could not be collected and analysed.

The choice of rapid systematic review protocol implied also to renounce a formal quality assessment of eligible articles. Therefore, we included only accepted articles, relying on the ability of the peer-review process of rejecting papers that did not meet the minimal criteria for publication. Since no meta-analysis was possible on the set of extracted data, the lack of a structured assessment of the risk of bias did not affect the final synthesis.

Somatic variants in the target tissues of CoVs were not comprised in the inclusion criteria of the present systematic search. The variable expression of virus receptors implies a complex network which controls expression level and processing in response to the virus, encompassing both coding and non-coding RNA molecules. The post-transcriptional regulation of the interaction between CoVs and the human host is a fascinating field of investigation—however, it was beyond the scope of our effort.

We weighted these limitations against the utility of a synopsis which summarises a heterogeneous and promising line of research. We are also aware that the body of evidence on the host genetic determinants of CoV-related phenotypes is increasing—our synthesis provides a snapshot of the current situation but is far from being conclusive. Due to the short time-lapse from the discovery of COVID-19, the majority of studies that were appraised in the present review are related to the SARS outbreak caused by SARS-CoV-1. This finding underlines the importance of reliable genetic association studies targeting the human host response to SARS-CoV-2 infection. In a rapidly changing scenario which has a major impact on public health, such as the current pandemic, it is mandatory to maintain the knowledge with the most recent evidence and discuss the research priorities. In adherence to the recommendations recently reminded by the Cochrane’s initiative [92], the review process outlined herein can be easily replicated in order to keep the scientific community abreast of the latest evidence.

### Lessons learned and perspectives

The considerations outlined above highlights the need for consortia aimed at coordinating multidisciplinary efforts to elucidate the genetic determinants of COVID-19 [15, 93]. To date, several dedicated programmes were launched within the European National Health Systems. The UK Biobank—which collects samples and detailed health data from about 500,000 volunteers—has started to analyse data collected from COVID-19 patients (https://www.ukbiobank.ac.uk/2020/04/covid/). In Finland, the COVID-19 Host Genetics Initiative intends to integrate data with major biobanks (https://www.covid19hg.org/). The Greek public initiative, COVID-19-GR, will genotype COVID-19 patients and their SARS-CoV-2 genome, linking data with the Greek COVID-19 registry (http://www.gsrt.gr/central.aspx?sId=119I428I1089I646I488772). In the UK, the GenOMICC Consortium is working in the project ‘GenOMICC study on COVID-19 patients’ aimed at achieving whole genome sequencing of up to 20,000 patients who were severely affected by COVID-19 and 15,000 who had mild symptoms (https://genomicc.org/). In Iceland, the company deCODE Genetics has partnered with Iceland’s government to sequence the genomes of patients who were previously infected with COVID-19 (https://www.decode.com/). In the USA, researchers from Harvard’s Wyss Institute and the Personal Genome Project at Harvard University are launching a multi-omics project to compare genomes, microbiomes, viromes, and immune systems of individuals with extreme susceptibility to SARS-CoV-2 infection and individuals who have shown resistance (https://wyss.harvard.edu). In collaboration with Yale University, the Yale SARS-CoV-2 Genomic Surveillance Initiative is using a variety of tools, including viral sequencing, case surveillance, test development, and wastewater surveillance, in order to monitor the spreading of the virus (https://covidtrackerct.com/). International initiatives, such as the COVID Human Genetic Effort [15] (www.covidhge.com), are having a pivotal role in facilitating the implementation of standardised phenotyping, collection of reliable clinical data, as well as biological samples, according to the best practices in adherence to shared ethical and legal standards. The GEFACOVID consortium, made up of 29 participating institutions, is focused on virus’ genetic polymorphisms and pathogenesis mechanisms as well as genetic, genomic, metabolomic, epidemiological and clinical data in order to identify biomarkers that confer susceptibility to virus infection and increase the risk of life-threatening complications (https://davincidtx.com/gefacovid-project/).

Registries and biobanks are invaluable sources of demographic and clinical assessments, including imaging and biomarkers. These large data sets may be used to explore different models of response to infection and generate hypotheses encompassing multiple genetic risk factors and interactions with other predictors. Promising hypotheses could be subsequently tested in clinical studies.

Assuming that ethnicity does not merely reflect the genetic background, as it is a complex construct made of genetic inheritance, cultural heritage and shared social and behavioural profiles, ancestry is a mandatory ascertainment in any clinical data set. Most genetic association studies included in the present review were targeted on patients of Chinese Han ancestry. In a global pandemic, the genetic diversity of populations across the world implies the need for inclusive investigations recruiting participants from the different genealogical and geographical origin. We endorse the recommendations recently invoked [94] and followed in an exploratory study [95], strongly suggesting the implementation of ethnicity forms and the analysis of data disaggregated by attributed ancestry.

## Conclusions

After the global spread of COVID-19, the knowledge about the interplay between CoVs and the human host has been growing at an impressive rate. However, as compared to the number of clinical studies that were delivered so far, we found a quite limited number of investigations focused on the effect of human genetic variability on clinical phenotypes.

A handful of candidate genes were tested in more than one study, and no single variant was confirmed to be associated with the clinical outcome in independent cohorts. The paucity of studies converging on common genetic determinants hampered any quantitative synthesis. In the light of the present synopsis, the discovery of a single major gene which determines a substantial variability of the CoV-associated phenotype cannot be sensibly anticipated. Since all genetic association studies eligible for the present review were presumably prone to both type I and type II errors, their findings should be regarded as exploratory.

Plenty of recent studies addressing the COVID-19 biological mechanisms and response to therapy might provide the rationale for novel candidate genes. Genetic association analyses warrant rigorous design and robust statistical methods, in order to test precise models of interaction among multiple genetic variants and between genetic variants and exposure. To this purpose, the shared effort of the human genomics community will enable the development of robust methodological approaches through a close multidisciplinary interchange, as well as the assembly of large data sets. Prompt dissemination of genetic studies—including negative findings—is helpful to focus on the rapidly evolving research questions.

Genetic variants are non-modifiable risk factors and cannot be claimed to be directly translated into the clinic as actionable markers in the present emergency. However, once a panel of associated genetic biomarkers is established, genetic biomarkers could be included in multidimensional models with age, comorbidities, socioeconomic status, etc., to prioritise public health interventions, such as active surveillance for the vulnerable population strata.

From the speculative perspective, although association studies do not prove mechanisms per se, evidence from genetic variants might be crucial to elucidate the biological pathways underlying the severe clinical presentation associated with CovS infection and to identify promising therapeutic targets.

## Supplementary information

**Additional file 1: Supplementary table 1.** Search strings used to interrogate publication databases. **Supplementary figure 1.** PRISMA workflow.

## Data Availability

All data relevant to the study findings were reported in figures and tables, either embedded in the manuscript or as supplementary material.
