# Supplementary material for: Genetic variants of the human host influencing the coronavirus-associated phenotypes (SARS, MERS and COVID-19): rapid systematic review and field synopsis
Source: Hum Genomics. 2020 Sep 11;14:30. doi: 10.1186/s40246-020-00280-6 (PMC7484929; doi:10.1186/s40246-020-00280-6)

Genetic variants of the human host influencing the coronavirus-associated phenotypes (SARS, MERS and COVID-19): rapid systematic review and field synopsis

Emilio Di Maria, Andrea Latini, Paola Borgiani, Giuseppe Novelli

SUPPLEMENTARY TABLE AND FIGURE

Supplementary table 1

Search strings used to interrogate publication databases.

Domain	MESH terms		Keywords	Filters
**GEN - Genetics**			genotype* OR polymorphi* OR allel* OR mutation* OR haploty* OR hla OR sequenc* OR genome-wide OR genomewide OR (genet* AND varia*) OR profil* OR expression OR mRNA OR miRNA OR microRNA	
**CoV - coronaviruses**	"Coronavirus/genetics" OR "SARS Virus"	OR	COVID-19 OR Coronavirus OR "Corona virus" OR "2019-nCoV" OR "SARS-CoV" OR "MERS-CoV" OR “Severe Acute Respiratory Syndrome” OR “Middle East Respiratory Syndrome”	
**CaB - CoV-associated Biomarkers**			ace2 OR ace-2 OR adam17 OR tmprss* OR c-type lectin OR CD209L OR L-SIGN OR il-4 OR interleukin-4 OR il-6 OR interleukin-6 OR mbl OR masp2 OR mxa OR tnf OR cd14* OR rantes	
**LAN - Language**				English
**YRS - Publication Years**				2003/01/01 – 2020/04/20<br>Update: 2020/04/20-2020/06/17

Supplementary figure 1

PRISMA workflow.

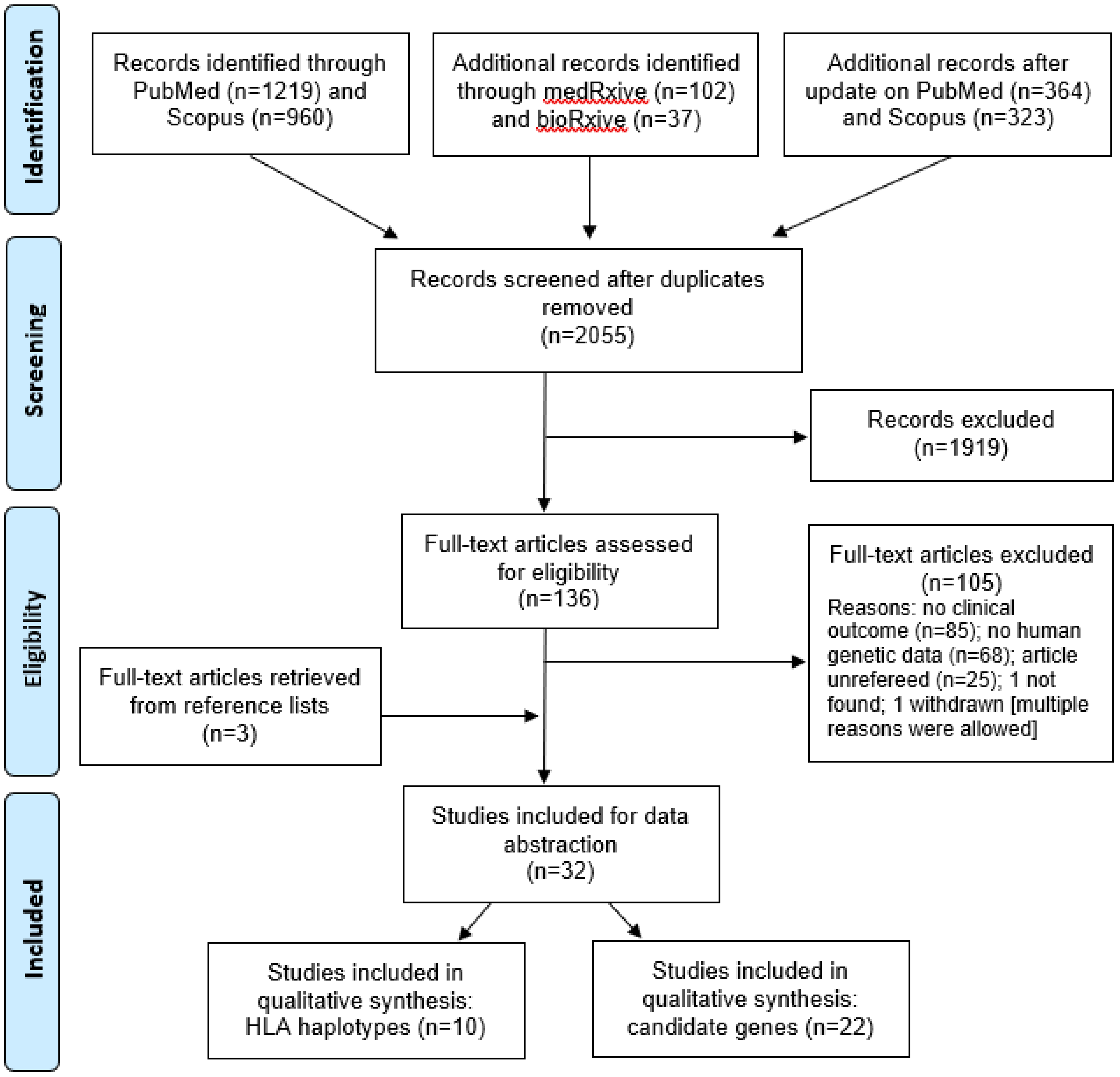

Supplement: Supplementary file 1 — Additional file 1: Supplementary table 1. Search strings used to interrogate publication databases. Supplementary figure 1. PRISMA workflow. [file 40246_2020_280_MOESM1_ESM.pdf]
